# Nuclear accumulation of host transcripts during Zika Virus Infection

**DOI:** 10.1371/journal.ppat.1011070

**Published:** 2023-01-05

**Authors:** Kristoffer E. Leon, Mir M. Khalid, Ryan A. Flynn, Krystal A. Fontaine, Thong T. Nguyen, G. Renuka Kumar, Camille R. Simoneau, Sakshi Tomar, David Jimenez-Morales, Mariah Dunlap, Julia Kaye, Priya S. Shah, Steven Finkbeiner, Nevan J. Krogan, Carolyn Bertozzi, Jan E. Carette, Melanie Ott

**Affiliations:** 1 J. David Gladstone Institutes, San Francisco, California, United States of America; 2 Department of Medicine, University of California, San Francisco, California, United States of America; 3 Medical Scientist Training Program, University of California, San Francisco, California, United States of America; 4 Biomedical Sciences Graduate Program, University of California, San Francisco, California, United States of America; 5 Stem Cell Program, Boston Children’s Hospital, Boston, Massachusetts, United States of America; 6 Department of Stem Cell and Regenerative Biology, Harvard University, Cambridge, Massachusetts, United States of America; 7 Division of Cardiovascular Medicine, Department of Medicine, Stanford University, Stanford, California, United States of America; 8 Departments of Chemical Engineering and Microbiology and Molecular Genetics, University of California, Davis, California, United States of America; 9 Center for Systems and Therapeutics and Taube/Koret Center for Neurodegenerative Disease Research, San Francisco, California, United States of America; 10 Departments of Neurology and Physiology, University of California, San Francisco, California, United States of America; 11 Quantitative Biosciences Institute (QBI), University of California, San Francisco, California, United States of America; 12 Department of Cellular and Molecular Pharmacology, University of California, San Francisco, California, United States of America; 13 Howard Hughes Medical Institute, Stanford University School of Medicine, Stanford, California, United States of America; 14 Department of Microbiology and Immunology, Stanford University, Stanford, California, United States of America; 15 Chan Zuckerberg Biohub, San Francisco, California, United States of America; University of Glasgow, UNITED KINGDOM

## Abstract

Zika virus (ZIKV) infects fetal neural progenitor cells (NPCs) causing severe neurodevelopmental disorders *in utero*. Multiple pathways involved in normal brain development are dysfunctional in infected NPCs but how ZIKV centrally reprograms these pathways remains unknown. Here we show that ZIKV infection disrupts subcellular partitioning of host transcripts critical for neurodevelopment in NPCs and functionally link this process to the up-frameshift protein 1 (UPF1). UPF1 is an RNA-binding protein known to regulate decay of cellular and viral RNAs and is less expressed in ZIKV-infected cells. Using infrared crosslinking immunoprecipitation and RNA sequencing (irCLIP-Seq), we show that a subset of mRNAs loses UPF1 binding in ZIKV-infected NPCs, consistent with UPF1’s diminished expression. UPF1 target transcripts, however, are not altered in abundance but in subcellular localization, with mRNAs accumulating in the nucleus of infected or UPF1 knockdown cells. This leads to diminished protein expression of FREM2, a protein required for maintenance of NPC identity. Our results newly link UPF1 to the regulation of mRNA transport in NPCs, a process perturbed during ZIKV infection.

## Author summary

The outbreak of Zika Virus (ZIKV) led to an epidemic of microcephaly and other congenital abnormalities. ZIKV is typically spread by mosquitoes, but it can also cross the placenta and infect the fetus of pregnant individuals. During the infection of the fetus, ZIKV targets neural progenitor cells (NPCs), leading to disruptions in brain development. In this study, we infect NPCs with ZIKV to better understand changes in the host cell during infection. In particular, we focus on the protein UPF1, a key player on the nonsense mediated mRNA decay pathway. We found that during ZIKV infection, UPF1 interacts with fewer transcripts, and these transcripts are associated with neurodevelopment. Furthermore, it appears that UPF1 is involved in mRNA transport out of the nucleus. We identify *FREM2* as one of the transcripts with the greatest loss of UPF1 interaction and find that perturbation of this gene leads to upregulation of proteins associated with neural progenitor cell differentiation. Our study provides new insights into both the molecular pathogenesis of ZIKV and highlights another role that UPF1 plays in the cell.

## Introduction

Zika virus (ZIKV) is a mosquito-borne, enveloped virus with a positive sense, single stranded RNA genome [1]. ZIKV is a member of the *Flaviviridae* family that includes Dengue virus (DENV), West Nile virus (WNV) and Hepatitis C virus (HCV). In 2015, an outbreak of ZIKV in Brazil was linked to a dramatic increase in the number of infants born with microcephaly [2,3]. It was subsequently shown that *in utero*, ZIKV infects neural progenitor cells (NPCs), resulting in neurodevelopmental delays that ultimately cause a range of birth defects including microcephaly, ocular damage, and contractures, collectively known as congenital Zika syndrome [4–8]. NPCs are critical for brain development as they differentiate into the glial and neuronal cells that compose the majority of the brain parenchyma [9]. Proposed molecular mechanisms by which ZIKV disrupts NPC function and differentiation focus on multiple cellular pathways including centrosomal organization, autophagy, apoptosis and unfolded protein response pathways [10–14]. However, it remains unknown how ZIKV manipulates multiple cellular pathways at once to cause widespread developmental reprogramming.

We and others previously showed that ZIKV infection suppresses the host nonsense-mediated mRNA decay (NMD) pathway [15,16]. NMD is an RNA quality control mechanism that targets faulty host transcripts for degradation and acts as an antiviral pathway on many viral species, particularly single-stranded RNA viruses [17,18]. Different models of the NMD pathway have been described, but the exon-junction complex (EJC)-mediated NMD pathway is the best defined [19,20]. This pathway is often triggered by premature termination codons in mRNAs [21].

The NMD pathway requires the recruitment of the RNA helicase and ATPase up-frameshift protein 1 (UPF1), which induces a cascade activating NMD-mediated degradation of faulty transcripts [22,23]. However, UPF1 is found to be associated with many transcripts [24], and the NMD pathway also regulates expression of non-faulty mRNAs, especially those with long and GC-rich 3’ untranslated regions (UTRs), a feature found in many viral RNAs [25–28]. UPF1 has been implicated in the regulation of a wide range of biological processes including other RNA decay pathways besides NMD [29], telomere maintenance [30], protein ubiquitination [31] and RNA export [32,33].

Because knockdown of UPF1 enhances replication of many viruses including ZIKV, WNV, DENV, Rous Sarcoma virus, Potato virus X, Pea Enation Mosaic virus 2, and Turnip Crinkle virus, NMD is recognized as a *bone fide* antiviral restriction pathway, often targeted by viruses including ZIKV for inactivation [17]. ZIKV inactivation of the antiviral activity of NMD involves the ZIKV capsid protein directly targeting UPF1 [15,16]. Similar interactions of capsid proteins with the NMD pathway also occur with other flaviviruses [16]. However, the finding that ZIKV capsid expression selectively downregulates UPF1 expression in the host nucleus was unexpected as viral RNA replication occurs exclusively in the host cytoplasm [15]. It remained unclear how downregulation of nuclear UPF1 supports viral replication.

Here we show that nuclear mRNA export is a UPF1 function that is disrupted during ZIKV infection. ZIKV infection or UPF1 knockdown resulted in polyadenylated transcript accumulation in the nucleus, causing decreased protein levels of FREM2, a UPF1 target, and consequent perturbation of NPC differentiation. We propose that by targeting nuclear UPF1 and trapping host mRNAs in the nucleus, ZIKV has evolved a mechanism to “shut off” host mRNA function while promoting translation of its own proteins. This mechanism describes a new central role for UPF1 in mRNA export connected to many cellular pathways associated with neurodevelopment.

## Results

### ZIKV infection decreases UPF1 interaction with the 3’UTR of host transcripts

**Fig 1:**
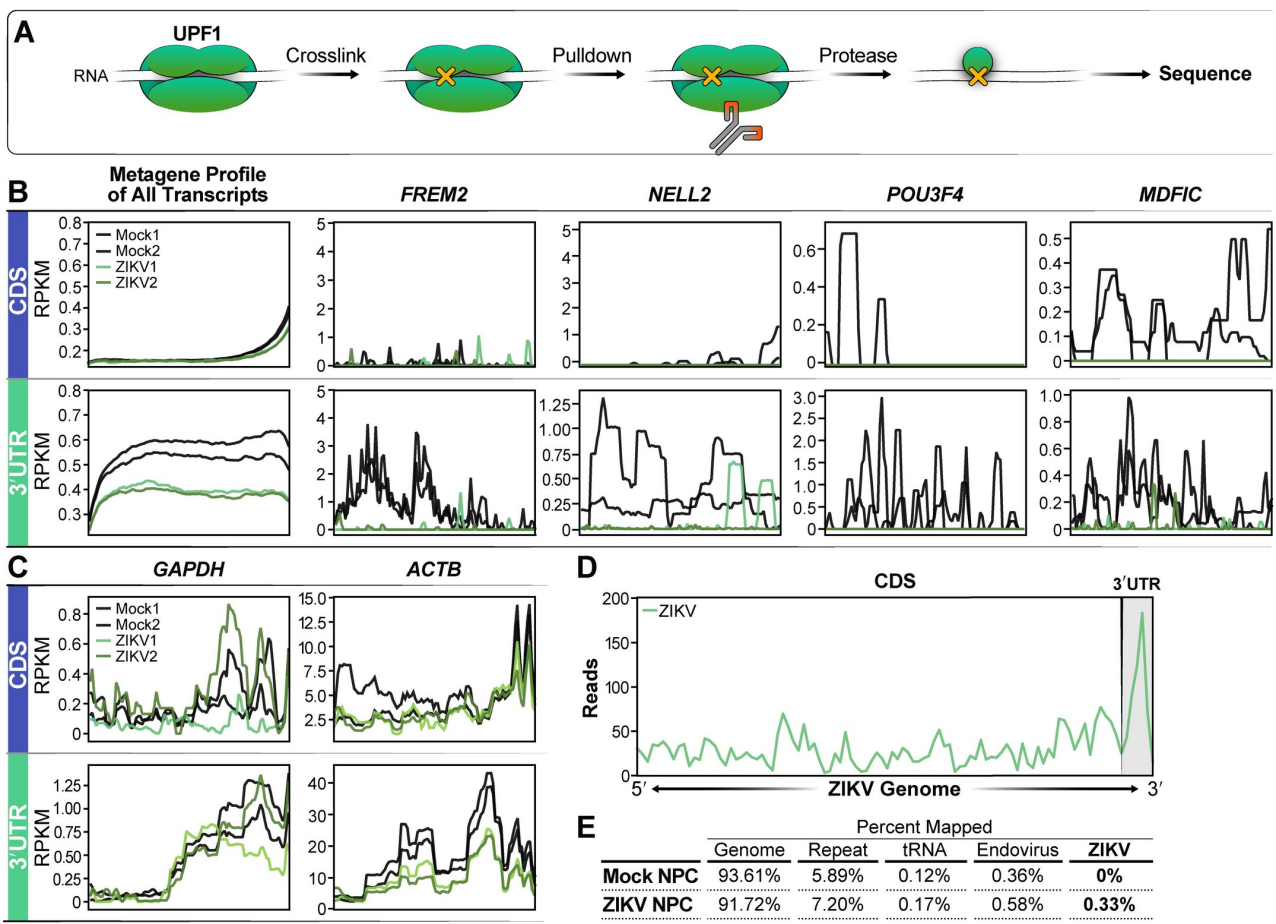
UPF1-host transcript interactions are decreased during ZIKV infection of NPCs. **A)** irCLIP schematic describing the workflow to obtain sequencing data. After 48 hrs of ZIKV infection, UPF1 and RNA are crosslinked using UV light, followed by UPF1 pulldown and protease degradation to expose the UPF1-bound RNA for sequencing. **B)** Metagene profile of all transcripts sequenced from the irCLIP experiment. The graphs show Reads Per Kilobase of transcript, per Million mapped reads (RPKM) values for positions in the Coding Domain Sequence (CDS) and the 3’UTR. Experiment was produced from 2 biological replicates. Representative metagene plots (FREM2, NELL2, POU3F4, MDFIC) of loci found to have a loss of UPF1 interaction. **C)** A metagene plot of GAPDH and ACTB are shown as controls. **D)** Metagene plot of reads mapping to the ZIKV genome, with the 3’UTR marked. **E)** Tabular breakdown of read map percentages from the UPF1-CLIP experiment.

To comprehensively define the UPF1-RNA interactions in human induced pluripotent stem cell-derived NPCs, we performed infrared crosslinking immunoprecipitation and RNA sequencing (irCLIP-Seq) [34]. NPCs were infected with ZIKV (isolate PRVABC59) at an MOI of 1 for 48 hours followed by UV-crosslinking of transcripts with proteins and immunoprecipitation of UPF1 (**Fig 1A**). We chose to use the Zika virus isolate PRVABC59, as it is a contemporary isolate from the ZIKV pandemic and has been previously associated with both neurodegeneration and NPC infection [35,36]. The infection rate of our NPCs was 9.3% and 12.1% for replicate 1 and 2 respectively. Mock-infected cultures served as controls. RNA-protein complexes were separated by SDS-PAGE, and mass spectrometry was performed on the excised bands to confirm UPF1 enrichment (**S1 Fig and S1 File**) before RNA was extracted and submitted to next-generation sequencing (**Fig 1A** and **S2 File**).

In mock-infected NPCs, UPF1 bound 6778 transcripts, predominantly at the 3’UTR, as previously reported [26,28]. In ZIKV-infected NPCs, this number decreased to 4557 transcripts, with a marked decrease in occupancy at the 3’UTR. A metagene profile of all transcripts as well as representative genes with the greatest loss of UPF1 interaction in the 3’UTR are shown (**Fig 1B**). Control transcripts such as glyceraldehyde-3-phosphate dehydrogenase (GAPDH) and Beta-Actin (ACTB) maintained UPF1 interaction (**Fig 1C**). The irCLIP data was compared to RNAseq data; this showed that there was overall less RNA, but notably, several transcripts that lost UPF1 binding did not show significant changes during ZIKV infection (**S2A Fig**). We performed an additional analysis of the irCLIP data focusing on the 3′UTR only that yielded an MA plot that identified transcripts that have both higher fold changes and appropriate abundances (**S2B Fig**).

In infected samples, UPF1 also interacted with ZIKV RNA, mostly within the viral 3’UTR, supporting the notion of an antiviral function of UPF1 through RNA binding and degradation (**Fig 1D**). We speculated that viral RNA could act as a “sponge” sequestering UPF1 away from host RNAs and explaining decreased UPF1 occupancy of host transcripts during infection. However, quantification of UPF1-bound reads mapping to host vs. viral RNAs showed that viral reads only accounted for 0.33% of all mapped reads while the majority of sequences isolated with UPF1 pulldown mapped to the host transcriptome. This excluded a “sponge” effect of viral RNA for UPF1 **(Fig 1E**).

### Selective reduction of UPF1 target transcripts in the cytoplasm of infected NPCs

**Fig 2:**
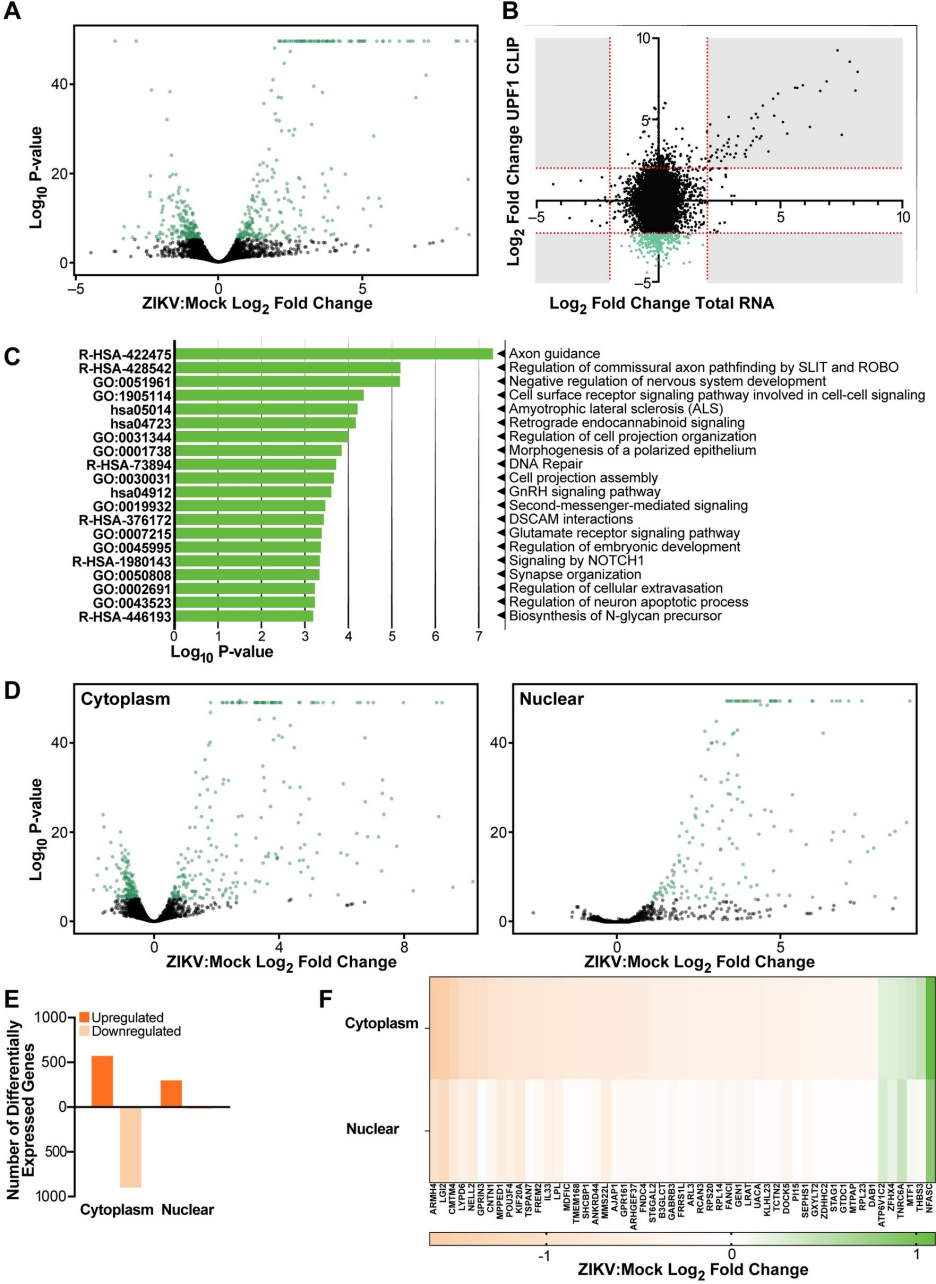
Loss of UPF1 interaction during ZIKV infection causes mRNA downregulation in the cytoplasm. **A)** Volcano plot of whole transcriptome RNA sequencing of ZIKV infected NPCs compared to Mock, n = 2 biological replicates.^#^ **B)** X-axis shows ZIKV: Mock log_2_ fold change in whole transcriptome sequencing, Y-Axis shows ZIKV:Mock log_2_ fold change from the UPF1 CLIP experiment. The green transcripts are >2 log_2_ fold change in the UPF1-CLIP and <2 log2 fold change in total RNA. **C)** Metascape analysis of transcripts identified to have a significant loss of UPF1 interaction in ZIKV infected cells compared to Mock. **D)** Volcano plot of sequencing from ZIKV-infected NPCs compared to Mock fractionated into cytoplasmic and nuclear fractions, n = 2 biological replicates.^#^ **E)** Number of differentially expressed genes from sequencing of ZIKV-infected NPCs compared to Mock fractionated into cytoplasmic and nuclear fractions **F)** Heatmap showing log_2_ fold changes of transcripts identified in the CLIP experiment in cytoplasmic and nuclear fractions. ^#^The upper limit of the Y-axis on the volcano plots was set to a value of 50 for consistency.

As UPF1 is central to NMD, we examined whether transcripts that lost UPF1 occupancy would be stabilized. For this, we performed whole transcriptome sequencing of ZIKV-infected NPCs followed by differential expression analysis (**S3 File**). Consistent with previous RNA-seq studies of ZIKV-infected NPCs [37,38], genes associated with the interferon response to infection were predominantly upregulated (**Figs 2A and S3B**). In contrast, the abundance of transcripts, which lost UPF1 occupancy upon infection, was not significantly changed (**Fig 2B and S2 File**). This indicates that likely only a fraction of UPF1-occupied transcripts is subject to degradation, underscoring the functional relevance of UPF1 functions outside of NMD.

Metascape analysis of transcripts with stable abundance after loss of UPF1 occupancy showed enrichment for neural and neurodevelopmental functions, such as axon guidance, highlighting the importance of UPF1 for neurodevelopment as previously indicated [39,40] **(Fig 2C**). NMD and UPF1 have been implicated in regulation of axon guidance, including specific localization patterns of NMD machinery in neuronal axons [41].

Next, we examined whether transcript localization was changed when UPF1 occupancy was lost. This was prompted by previous reports that indicated that UPF1 translocates between the nucleus and cytoplasm [42], and our previous observation that UPF1 is selectively downregulated in the nucleus upon infection [15]. ZIKV- and mock-infected NPCs were fractionated into cytoplasmic and nuclear compartments, and RNA sequencing followed by differential expression analysis was performed for each fraction (**S4 and S5 Files)**.

We confirmed successful fractionation by comparing the log_2_ fold change of known cytoplasmic (*GAPDH*) and nuclear (*ANRIL*) transcripts. *GAPDH* was 3.2-fold enriched in the cytoplasmic fraction and ANRIL 2.3-fold in the nuclear fraction, as expected (**S3A Fig**). Upon ZIKV infection, we identified 585 and 312 significantly upregulated mRNAs in the cytoplasm and nucleus, respectively (**Fig 2C**). The upregulated transcripts in the whole cell and in the fractionated compartments were predominantly interferon response genes (**S3B and S3C Fig**). In contrast, downregulated transcripts were overwhelmingly cytoplasmic, with 912 mRNAs showing less abundance in the cytoplasm compared to 22 in the nuclear fraction (**Fig 2E**). Moreover, due to the dataset size, we used a strict cutoff of a log_2_ fold change of at least 2 to identify the top mRNAs with the most significant decrease in UPF1 interaction upon infection. When we examined these transcripts in the fractionated sequencing experiment, all but 6 were downregulated in the cytoplasm (**Fig 2F**). These results show a selective loss of UPF1 target mRNAs in the cytoplasm of infected cells. As total abundance of these transcripts was unchanged, we considered the possibility that they may accumulate in the nucleus upon ZIKV infection.

### Nuclear accumulation of host transcripts upon ZIKV infection and UPF1 knockdown

**Fig 3:**
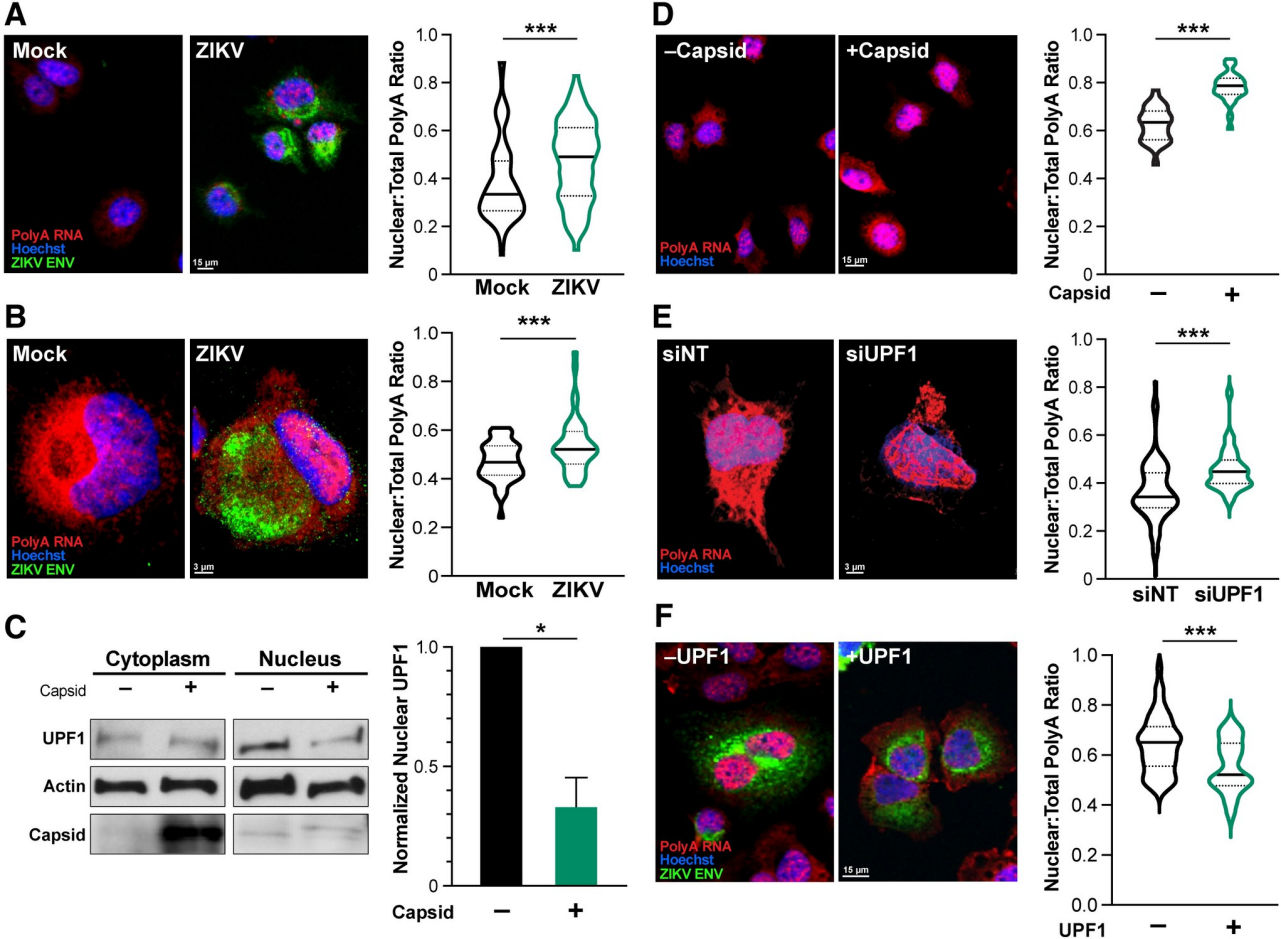
ZIKV-mediated degradation of UPF1 leads to mRNA retention in the nucleus. **A and B)** During ZIKV infection (ZIKV ENV, green), polyA RNA (red) is shifted toward the nucleus (Hoechst, blue) in Huh7-Lunet cells (A) and NPCs (B). Statistics produced by a Linear Mixed Model. 3 biological replicates, n = 25 cells (A) or 15 cells (B) per condition per replicate. **C)** Tetracycline-inducible Capsid expression in Huh7-Lunet cells was used to degrade nuclear UPF1. Leptomycin B (LMB) was used to increase UPF1 degradation. Statistics performed by Student’s t-test, n = 3, and representative western blot shown. **D)** Capsid overexpression in Huh7-Lunet cells results in an increased ratio of polyA RNAs (red) in the nucleus. N = 3 biological replicates, 25 cells per condition per replicate. **E)** Knockdown of UPF1 in NPCs results in an increased ratio of polyA RNAs (red) in the nucleus. NPCs were treated with siNT and siUPF1 for 96 hours. Statistics produced by a Linear Mixed Model. n = 3 biological replicates, 15 cells per condition per replicate. **F)** Tetracycline-inducible UPF1 expression in Huh7-Lunet cells was used in conjunction with ZIKV infection to prevent the polyA RNA shift to the nucleus. Statistics produced by a Linear Mixed Model. n = 3 biological replicates, 15 cells per condition per replicate. *, P ≤ 0.05; **, P ≤ 0.01; ***, P ≤ 0.001. Error bars are SEM. Individual channels are shown in S5 Fig. Nuclear to cytoplasmic ratios are provided in S6 File.

To test the hypothesis that transcripts were selectively accumulating in the nucleus in ZIKV-infected cells, we analyzed global transcript localization using RNAscope analysis with a polyA tail probe. We first optimized the technology in Huh7-Lunet cells, a hepatoma cell line frequently used to study flavivirus infection [43], and then in NPCs, both infected with ZIKV at an MOI of 1. Confocal microscopy was used to determine fluorescence, as described in the Materials and Methods section. The magnification used during imaging was cell type-specific, with 20x used for Huh7 cells and 63x used for NPCs, due to the differences in cell size. The imaging software package Imaris was used to bound cellular compartments and calculate fluorescence values using their proprietary tools. In both cell types, polyadenylated RNA fluorescence was significantly increased in the nucleus upon infection, supporting the model that mRNA location rather than abundance is perturbed in ZIKV-infected cells (**Fig 3A and 3B**).

To determine if this effect was UPF1-mediated, we leveraged the previously demonstrated ability of ZIKV capsid to specifically degrade nuclear UPF1 [15], and used Huh7-Lunet cells conditionally expressing ZIKV capsid protein. Induction of capsid expression by addition of doxycycline led to reduced levels of nuclear UPF1 (**Fig 3C**). Capsid expression also led to an accumulation of polyadenylated RNAs in the cell nucleus, mirroring the effect observed in ZIKV-infected cells (62% nuclear fluorescence in vector vs 78% in capsid overexpressing cells) (**Fig 3D**).

Furthermore, siRNA-mediated knockdown of UPF1 in NPCs also resulted in retention of polyadenylated RNAs in the nucleus, underscoring the role of UPF1 in this process (36% nuclear fluorescence in control vs 46% in siUPF1 NPCs) (**Figs 3E and S4A**). To determine whether the loss of UPF1 controls mRNA accumulation in ZIKV-infected cells, we generated Huh7-Lunet cells conditionally overexpressing UPF1 and infected them with ZIKV. UPF1 overexpression decreased the ZIKV-induced shift of polyadenylated RNAs toward the nucleus, functionally linking UPF1 expression and nuclear mRNA accumulation (64% nuclear fluorescence in infected control vs 55% in infected UPF1 overexpressing cells) (**Figs 3F** and **S4B**). Collectively, these data uncover a new function of UPF1 in mRNA transport in NPCs, a function perturbed upon loss of UPF1 expression during ZIKV infection.

### UPF1 regulates *FREM2* mRNA localization, FREM2 protein expression, and NPC differentiation

**Fig 4:**
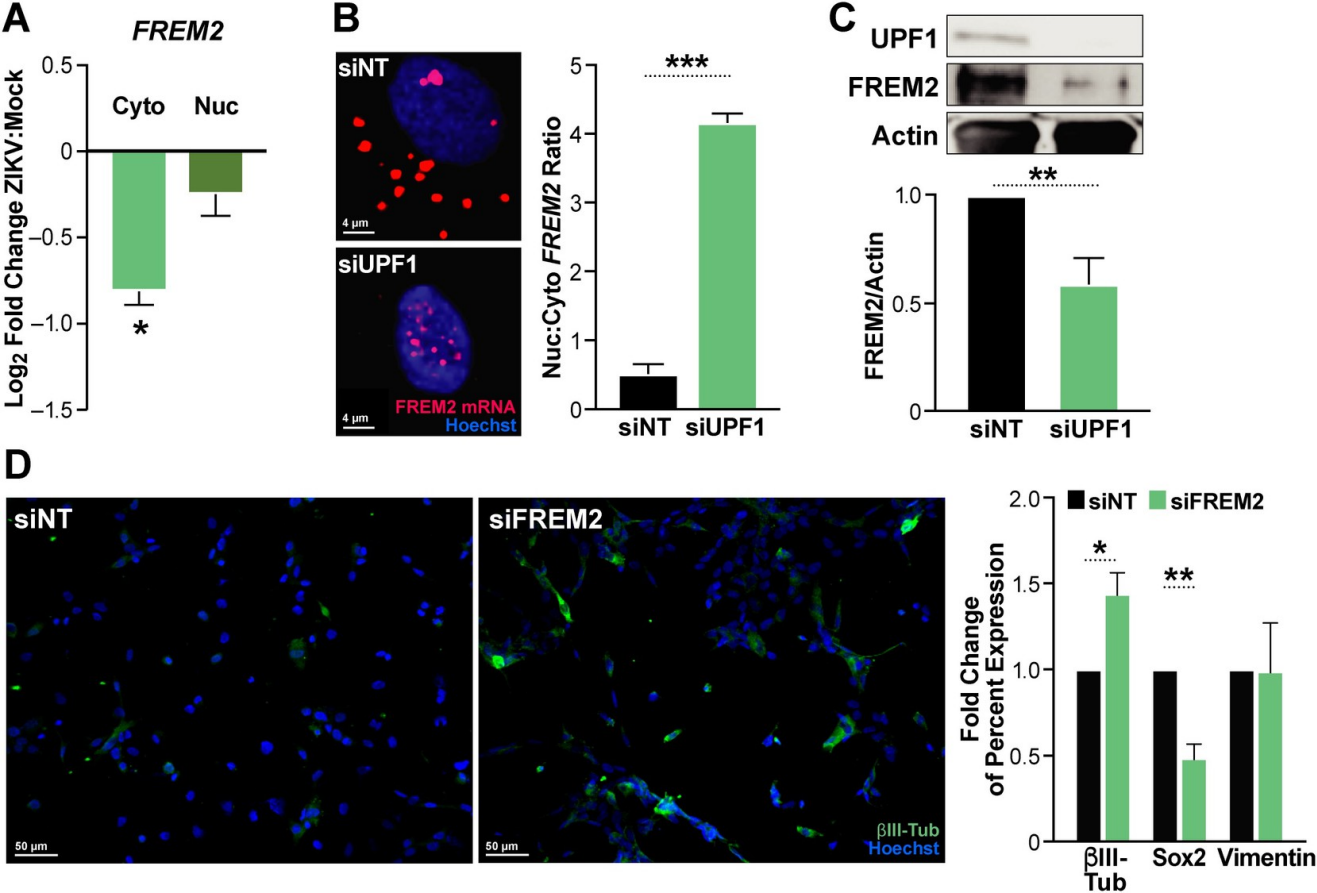
UPF1 knockdown leads to retention of *FREM2* mRNA and decreased protein production, which alters differentiation markers. **A)** RT-qPCR of *FREM2* in cytoplasmic and nuclear fractions in ZIKV-infected NPCs. **B)** RNAscope of *FREM2* in UPF1 knockdown NPCs. NPCs were treated with siRNAs for 96 hours prior to harvesting for microscopy. Number of nuclear puncta compared to cytoplasmic as calculated by Imaris. 3 biological replicates, 10 cells per biological replicate per condition averaged. Statistics produced by Student’s t-test. **C)** Western blot of siNT and siUPF1 treated NPCs. NPCs were treated with siRNAs for 96 hours prior to harvesting for microscopy. Densitometric analyses of FREM2 were performed using ImageJ to quantify relative band intensities. **D)** siNT and siFREM2 treated NPCs stained for βIII-Tubulin, Sox2 and Vimentin. NPCs were treated with siRNAs for 7 days prior to analysis. Statistics produced by Student’s t-test. *, P ≤ 0.05; **, P ≤ 0.01; ***, P ≤ 0.001. Error bars are SEM. See S8 Fig for multi-channel images and S9 Fig for the western blots used.

Next, we focused on *FREM2*, the top transcript with the largest fold decrease in UPF1 interaction in the irCLIP studies (**Fig 1B**). FREM2 is an extracellular matrix protein involved in cell-cell interactions that is important in many developmental pathways including tissue and vascular morphogenesis [44,45]. The *FREM2* transcript was specifically downregulated in the cytoplasm of infected NPCs in the fractionated mRNA sequencing analysis (**Fig 2F**). We confirmed selective cytoplasmic downregulation of *FREM2* mRNA expression upon infection by cell fractionation and RT-qPCR (**Fig 4A**). Using RNAscope, we found enrichment of the *FREM2* transcript in the nucleus of cells treated with UPF1-targeting siRNAs as compared to cells treated with non-targeting control siRNAs, where it was found in both the cytoplasm and the nucleus (**Fig 4B**). Consequently, FREM2 protein levels were significantly decreased in UPF1 siRNA-treated cells (42% decrease in siUPF1 compared to the siNT control), supporting the model that UPF1 posttranscriptionally regulates expression of critical neurodevelopmental genes, such as *FREM2*, through control of nuclear:cytoplasmic mRNA transport. These observations also support a “host shut-off” model where nuclear mRNA retention leads to reduced translation of UPF1 target transcripts during ZIKV infection (**Fig 4C**).

To determine the functional consequences of reduced FREM2 expression, we used siRNAs to knockdown FREM2 in NPCs (**S6 Fig**). FREM2 knockdown increased the percentage of cells expressing the neuronal lineage marker βIII-Tubulin by 44%, and decreased the percentage of cells expressing the pluripotency marker Sox2 by 51% (**Fig 4D**). The increase in βIII-Tubulin as a marker of neuronal differentiation also occurs during ZIKV infection of human NPCs (**S7 Fig**) [46]. These results indicate that loss of FREM2 promotes premature neuronal differentiation of NPCs, theoretically reducing the number of proliferating NPCs required for appropriate development of the brain.

## Discussion

In this study, we describe a potential host “shut-off” mechanism by which ZIKV infection prevents host mRNA export from the nucleus, a phenotype described in other viral infections, but not seen previously with flaviviruses [47–50]. Many transcripts lost UPF1 occupancy during ZIKV infection, especially in the 3’UTR, and this loss of UPF1 was associated with altered transcript localization and only a minor effect on transcript abundance. This underscores the significance of UPF1 function outside its well-described role in the NMD pathway.

The majority of these experiments were performed in human NPCs, the natural target cells of ZIKV in fetuses, providing a physiological context for the pathways and transcripts disrupted by ZIKV infection and potentially contributing to congenital Zika syndrome. A caveat of NPC infections with PRVABC59 is their low infection efficiency [35]. Accordingly, we found for an MOI of 1 an infection rate of approximately 10% in NPC infections. Therefore, bystander effects can contribute to phenotypes obtained in bulk assays such as irCLIP and RNA-Seq but not in single-cell assays such as RNAScope and immunofluorescence, which differentiate between infected and uninfected cells. Bystander effects include interferon-response signaling pathways, non-productive ZIKV infections or translocation of ZIKV capsid to adjacent cells [51]. Notably, the contemporary isolate of ZIKV used in this study is known to cause congenital Zika syndrome [52]. In a previous study, we showed that capsid from the African lineage as well as the contemporary strain of ZIKV both interact with UPF1 [15]. Therefore, our findings may not be unique to the contemporary strain of ZIKV.

In this study, the accumulation of mRNAs in the nucleus induced by ZIKV infection could be recapitulated both by ZIKV capsid expression, which is known to degrade nuclear UPF1, and by UPF1 knockdown, implicating UPF1 and possibly its nuclear localization in this function. Importantly, overexpression of UPF1 reversed nuclear mRNA accumulation during ZIKV infection, confirming the functional relevance of UPF1 in the observed viral phenotype. Lastly, we show that when UPF1 is unable to export *FREM2* mRNA from the nucleus, FREM2 protein abundance decreases, supporting the model of a host shut-off mechanism where decreased translation of cellular mRNAs potentially enables more efficient viral translation and propagation.

Two other studies have linked UPF1 to nuclear export. The first study found that HIV-1 required UPF1 to export viral RNA from the nucleus of HeLA cells [33]. The second study found that UPF1 translocates between the nucleus and cytoplasm in *Drosophila*, and UPF1 depletion disrupts mRNA export from the nucleus [32]. The finding that ZIKV has evolved a unique function for its capsid protein to interfere with UPF1’s role in human mRNA export underscores the central role of UPF1 in this process, at least in the two cell types (Huh7 and NPCs) that we studied here. How this function is regulated and whether it involves known UPF1 interacting proteins such as the EJC or known RNA export pathways such as CRM-1, remains to be determined.

While the precise mechanisms of UPF1-mediated nuclear export are not yet clear, two findings underscore its significance: 1) most transcripts were not upregulated in NPCs after loss of UPF1 interaction, which points to a major function of UPF1 in cells other than NMD, and 2) many polyadenylated transcripts were retained in the nucleus upon UPF1 knockdown, demonstrating that the effect of UPF1 on mRNA export is widespread. Additional studies are needed to determine whether specific features of these mRNAs, such as the 3’UTR sequence or structure, underlie their targeting for export by UPF1 [53]. However, during ZIKV infection, many different interactions exist between viral and host proteins, especially as it pertains to the nuclear pore complex [54,55]. While we propose that UPF1 is one player in the widespread dysregulation of these interactions, further studies are needed to elucidate disruptions in nuclear translocation during viral infection.

Our studies confirm that UPF1 binds ZIKV RNA consistent with its restrictive role on viral replication [15]. Here, we map this binding to the ZIKV 3’UTR, similar to what is observed with host mRNAs. Interestingly, a non-coding subgenomic flavivirus RNA (sfRNA) is derived from the 3’ UTR of ZIKV and is known to be resistant to XRN1 degradation [56]. The production of sfRNA is shared among flaviviruses and antagonizes the interferon response [57]. Our data are consistent with UPF1 potentially regulating sfRNA production or interacting with ZIKV sfRNAs, which could affect ZIKV replication independently from NMD.

We speculate that the widespread nuclear retention of host mRNAs caused by the disruption of UPF1 function could be part of a central mechanism explaining pleiotropic effects of ZIKV infection on diverse cellular pathways. We pursued *FREM2* as our top UPF1 target, and show that it is downregulated at the protein level when UPF1 is unable to perform its role as a nuclear mRNA export regulator. FREM2 is both a member of the FRAS/FREM complex and regulates its formation [44]. The FRAS/FREM complex is found in cellular basement membranes and is expressed differentially during development [45]. FREM2 itself is important for proper development of the eye and FREM2 mutation is associated with Fraser syndrome, in which cryptopthalamos (a congenital defect where the eyes are covered completely by skin and often associated with small or missing eyeballs) is commonly seen [58]. Ophthalmologic manifestations are seen in up to half of children with congenital Zika syndrome, supporting the possibility that retention of the *FREM2* transcript in the nucleus could be directly involved in pathogenesis [59,60].

Nuclear retention is a new host shutoff mechanism previously not described for flaviviruses. Flaviviruses, including ZIKV, have been reported to cause host shutoff by translational repression [61,62]. ZIKV establishes a productive infection within 24 hours, rapidly causing perturbations in cellular function including protein synthesis shutoff during this time [62,63]. Importantly, a number of different pathways beyond apoptosis have been implicated in disruption of neurogenesis during ZIKV infection, including the innate immune response, unfolded protein response, centrosomal defects, and autophagy [64–67]. We believe that mRNA export defects are another potential mechanism contributing to the phenomenon of neuropathogenesis.

We confirm that a single viral protein, the capsid protein, is sufficient to degrade nuclear UPF1 and cause nuclear mRNA retention. Notably, capsid is one of few ZIKV proteins that can localize to the cell nucleus [68], which is independent from its function as a structural component of the virion [69]. Our study highlights a possibly important role for nuclear UPF1, which has been associated with cell cycle progression, DNA replication, telomere maintenance, and mRNA release [32,42] but is overall less well studied than the cytoplasmic form. The finding that ZIKV has evolved a mechanism to selectively target nuclear UPF1 and also disrupt its mRNA export function underscores the significance of the nuclear form of UPF1 in viral defense and potential impacts on neural development. Lastly, the significance of these findings may have broader implications for the biology of virally induced pathologies. Diverse viruses are known to interact with UPF1 and the NMD pathway [17], and our findings add additional understanding of the level and mechanism of UPF1 dysregulation, specifically in the developing brain.

## Materials and methods

### Cell culture and viruses

Human iPSC-derived NPCs were generated and maintained as described previously [70]. The human fibroblast cell line used to generate iPSCs came from the Coriell Institute for Medical Research and Yale Stem Cell Center. The iPSCs used in these studies was the CTRL2493n17 line. CTRL2493n17 was derived from the parental fibroblast line ND31845 that was biopsied from a healthy female at 71 years of age. iPSCs were cultured and maintained in complete mTESR (StemCell Techologies, Vancouver, CA). NPCs were differentiated and maintained using growth factor enriched EFH media composed of Stemline Neural Stem Cell Media (Sigma Aldrich, St. Louis, MO), EGF (R&D Biosystems, Minneapolis, MN), rhFGF basic (R&D Biosystems), and Heparin Sulfate (Sigma Aldrich). NPCs were dissociated and plated onto Matrigel-coated plates (Corning, Corning, NY) prior to infecting with ZIKV (MOI of 1) or treating with siRNAs. Experiments were harvested 48 hours post infection and up to 7 days after siRNA treatment. Huh7-Lunet cells (Ralf Bartenschlager, Heidelberg University), and Vero cells (ATCC CCL-81) were maintained in Dulbecco’s modified Eagle’s medium (DMEM) with 10% fetal bovine serum (FBS), 2 mM l-glutamine, 100 U/ml penicillin, and 100 μg/ml streptomycin.

Sequences encoding for N-terminally Strep TagII tagged ZIKV Capsid was cloned to pLVX-TetOne-Puro Vector (Clontech, Mountainview, CA, Cat: 631849) using BamHI & EcoRI cut sites. psPAX2 and pMD2.G were a gift from Didier Trono (Addgene plasmid # 12260 and # 12259 respectively); pCW57.1-Tet-UPF1WT (pRKB264) was a gift from Robert Bradley (Addgene plasmid # 99146) [31]. Tetracycline inducible cell lines were generated by using a 2nd generation lentivirus system. Briefly, 293T cells (ATCC CRL-3216) were transfected with plasmid DNA. Supernatant containing pseudovirus particles was collected at 48 hours post transfection, filtered and used to transduce Huh7-Lunets overnight with polybrene. After transduction, the Huh7-Lunets were selected with puromycin for a minimum of 7 days. Expression of either ZIKV capsid or UPF1 was confirmed by western blot after addition of doxycycline.

The strain PRVABC59 of ZIKV (ATCC, Manassas, VA, VR-1843) was used for all experiments. ZIKV stocks were propagated in Vero cells (ATCC CCL-81), and titers were determined by plaque assays on Vero cells. ZIKV infections were performed by adding viral inoculum to DMEM with 2% FBS or EFH followed by a two-hour incubation at 37C with a rock every 15 minutes. After infection was completed, inoculum was aspirated and then fresh DMEM with 10% FBS or EFH was added to the cells. Infected cells were cultured for 48 hours prior to harvesting for all sequencing and IF experiments.

### Antibodies and other reagents

Primary antibodies used were anti-UPF1 (Bethyl laboratories, Montgomery, TX, A300-38A, CST, Newburyport, MA, 12040S and Abcam, Cambridge, UK, ab109363), anti-FREM2 (Invitrogen, Carlsbad, CA, PA5-20982), anti-FLAG (Abcam, ab18230), anti-Actin (CST, 4967S), anti-GFAP (Abcam, ab53554), anti-Nestin (Abcam, ab22035) and anti-Beta III tubulin (Abcam, ab18207). Secondary antibodies used include goat anti-rabbit Alexa 488 (Invitrogen, A-11008), goat anti-mouse Alexa 488 (Invitrogen, A-11001), goat anti-rabbit Alexa 594 (Invitrogen, A-11012), goat anti-mouse Alexa 594 (Invitrogen, A-11005), donkey anti-goat Alexa 647 (Invitrogen, A-21447), donkey anti-rabbit Alexa 488 (Invitrogen, A-21206), donkey anti-mouse Alexa 594 (Invitrogen, A-21203). The RNAScope Multiplex Fluorescent V2 Assay (ACD, Newark, CA, 323100) was used with RNAscope Probes include polyA RNA (ACD, 318631) and FREM2 (ACD, 482841). Opal570 (Akoya Biosciences, Marlborough, MA, FP1488001KT) was used for visualization of RNAscope probes. SMARTpool Accell siRNA, which utilizes 4 siRNAs, was used for knockdown of FREM2 (Dharmacon, Lafayette, CO, E-021693-00-0010) and UPF1 (Dharmacon, E-011763-00-0010) according to manufacturer’s instructions. Non-targeting siRNAs used were cat. D-001910-10-20 (Dharmacon). Huh7-Lunets were treated with 60 ng/mL of Leptomycin B (Cayman Chemical) for 16 hours.

### Infrared crosslinking and immunoprecipitation

irCLIP was performed as in Zarnegar et al. 2016. Cells grown as described above and UV crosslinked to a total of 0.35 J/cm^2^. Whole-cell lysates were generated in CLIP lysis buffer (50 mM HEPES, 200 mM NaCl, 1 mM EDTA, 10% glycerol, 0.1% NP-40, 0.2% Triton X-100, 0.5% N-lauroylsarcosine) and briefly sonicated using a probe-tip Branson sonicator to solubilize chromatin. Each experiment was normalized for total protein amount, typically 1 mg, and partially digested with RNase A (ThermoFisher Scientific, Waltham, MA, EN0531) for 10 minutes at 37°C and quenched on ice. UPF1 (Bethyl laboratories, A300-38A) IP’s were performed using 15 μg of each antibody with 50 μL Protein G Dynabeads (ThermoFisher Scientific), for 8 hours at 4°C on rotation. Samples were washed sequentially in 1 mL for 1 minute each at 25°C: 1× high stringency buffer (15 mM Tris-HCl, pH 7.5, 5 mM EDTA, 2.5 mM EGTA, 1% Triton X-100, 1% sodium deoxycholate, 120 mM NaCl, 25 mM KCl), 1× high salt buffer (15 mM Tris-HCl pH 7.5, 5 mM EDTA, 2.5 mM EGTA, 1% Triton X-100, 1% sodium deoxycholate, 1 M NaCl), 2× NT2 buffer (50 mM Tris-HCl, pH 7.5, 150 mM NaCl, 1 mM MgCl_2_, 0.05% NP-40). After the NT2 wash, RNA-protein complexes were dephosphorylated with T4 PNK (NEB) for 45 minutes in an Eppendorf Thermomixer at 37°C, 15 seconds 1400rpm, 90 seconds of rest in a 30 μL reaction, pH 6.5, containing 10 units of T4 PNK, 0.1 μL SUPERase-IN (ThermoFisher Scientific), and 6 μL of PEG-400 (16.7% final). Dephosphorylated RNA-protein complexes were then rinsed once with NT2 buffer and 3’-end ligated with T4 RNA Ligase 1 (NEB, Ipswich, MA) overnight in an Eppendorf Thermomixer at 16°C, 15 seconds 1400rpm, 90 seconds of rest in a 60 μL reaction containing 10 units T4 RNA Ligase, 1.5 pmol pre-adenylated-IR800-3’biotin DNA-adapter, 0.1 μL SUPERase-IN, and 6 μL of PEG400 (16.7% final). The following day, samples were again rinsed once with 500 μL NT2 buffer and resuspended in 30μL of 20 mM DTT, 1x LDS (ThermoFisher Scientific) in NT2 buffer. Samples were heated to 75°C for 10 min, and released RNA-protein complexes were separated on 4–12% Bis-Tris Gels (1.0mm X 12 well) at 200V for 45 min. Resolved RNP complexes were wet-transferred to nitrocellulose at 550 mA for 45 minutes at 4°C.

Nitrocellulose membranes were imaged using an Odyssey CLx scanner (LiCor, Lincoln, Nebraska), RBP-RNA complexes were excised using scalpels, and RNA was recovered by adding 0.1 mL of Proteinase K reaction buffer (100 mM Tris, pH 7.5, 50 mM NaCl, 1 mM EDTA, 0.2% SDS) and 5 μL of 20mg/mL Proteinase K (ThermoFisher Scientific). Proteins were digested for 60 minutes at 50°C in an Eppendorf Thermomixer. Next, 200 μL of saturated-phenol-chloroform, pH, 6.7 was added to each tube and incubated for 10 minutes at 37°C in an Eppendorf Thermomixer, 1400 rpm. Tubes were briefly centrifuged and the entire contents transferred to a 2 mL Heavy Phase Lock Gel (5Prime, South San Francisco, CA, 2302830). Samples were centrifuged for 2 minutes at >15600 x g. The aqueous layer was re-extracted with 1 mL of chloroform (inverting 10 times to mix; no vortexing) in the same 2 mL Phase Lock Gel tube and centrifuged for 2 minutes at >15600 x g. The aqueous layer was then transferred to a new 2 mL Heavy Phase Lock Gel tube and extracted again with an additional 1 mL of chloroform. After 2 minutes centrifugation at >13000 rpm, the aqueous layer was transferred to a siliconized 1.5 mL tube and precipitated overnight at -20°C by addition of 10 μL 5M NaCl, 3 μL Linear Polyacrylamide (ThermoFisher Scientific) and 0.8 mL 100% ethanol. RNA fragments were pelleted at 15600 x g for 45 minutes at 4°C, washed once with 1 mL of ice cold 75% ethanol and air dried.

RNA pellets were resuspended in 12 μL water. 1 μL of 3 μM cDNA and 1 μL of 10mM dNTPs and heated to 70°C for 5 minutes then rapidly cooled to 4°C. cDNA Master Mix (4 μL 5x Super Script IV (SSIV) Buffer, 1 μL 100mM DTT, 1 μL SSIV, 6 μL total) was added to the annealed RNA and incubated for 30 minutes at 55°C. cDNA:RNA hybrids were captured by addition of 5 μL of MyOne Streptavidin C1 Dynabeads (ThermoFisher Scientific) that had been rinsed and suspended in 50 μL of Biotin-IP buffer (100mM Tris, pH 7.5, 1M NaCl, 1mM EDTA, 0.1% Tween), and end over end rotation for 45 minutes at room temperature. Beads were placed on a 96-well magnet and washed sequentially twice with 100 μL of Biotin IP buffer and 100 μL ice-cold 1xPBS. Beads were resuspended in 10 μL of cDNA elution buffer (8.25 μL water, 1 μL of 1 μM P3 short oligo, and 0.75 μl of 50 mM MnCl_2_) and heated to 95°C for 10 minutes, ramp 0.1 degree/second to 60°C forever. Next 5 μL of circularization reaction buffer was added (3.3 μL water, 1.5 μL 10x Circligase-II buffer, and 0.5 μL of Circligase-II (Epicentre, Madison, WI)). cDNA was circularized for 2 hours at 60°C. cDNA was purified with 30 μL of AMPure XP beads (Beckman Coulter, Pasadena, CA) and 75 μL of isopropanol. Samples were incubated for 20 minutes at 25°C, washed twice with 100 μL 80% ethanol, air dried for 5 minutes, and eluted in 14 μL of water. Elution took place at 95°C for 3 minutes and the eluent was immediately transferred to a 96-well magnet. Eluted cDNA was transferred to a new PCR tube containing 15 μL of 2X Phusion HF-PCR Master Mix (NEB), 0.5 μL of 30 μM P3/P6 PCR1 oligo mix and 0.5 μl of 15x SYBR Green I (ThermoFisher Scientific). Real-time quantitative PCR was performed: 98°C 2 min, 15 cycles of 98°C 15 seconds, 65°C 30 seconds, 72°C, 30 seconds, with data acquisition set to the 72°C extension. PCR1 reactions were cleaned up by adding of 4.5 μL of isopropanol, 54 μL of AMPure XP beads and incubation for 10 min. Beads were washed once with 80% ethanol, dried for 5 min, and eluted in 15 μl of water. Illumina flow cell adaptors were added by adding 15 μL 2X Phusion HF-PCR Master Mix and 0.4 μL P3solexa/P6solexa oligo mix and amplified: 98°C 2 min, 3 cycles of 98°C 15 seconds, 65°C 30 seconds, 72°C, 30s seconds. Final libraries were purified by addition of 48 μL of AMPure XP beads and incubation for 5 min. Beads were washed twice with 70% ethanol, dried for 5 min, and eluted in 20 μL of water. 1–2μL of libraries were quantitated by HS-DNA Bioanalyzer. Samples were deep sequenced on the Illumina NextSeq machine: single-end, no index, high-output, 75-bp cycle run.

Whole transcriptome RNA sequencing was performed using the methods described above for RNA extraction, library preparation and sequencing.

### Nuclear/cytoplasmic RNA fractionation and sequencing

Fractionation was performed using the Cytoplasmic and Nuclear RNA Purification Kit (Cat. # 2100, Norgen Biotek Corp, Thorold, CA). Purified RNA was treated with DNase I followed by library preparation using the NuGEN V2 RNA-Sequencing Library Preparation kit (Tecan Genomics, Redwood City, CA). Both RNA and library quality were analyzed via a Bioanalyzer (Agilent, Santa Clara, CA). Sequencing was performed on a NextSeq 500 (Illumina, San Diego, CA): single-end, no index, high-output, 75-bp cycle run.

### IF and RNAscope protocol

For immunofluorescence and RNAscope, infected NPCs were collected at 48 hours and plated onto 22- by 22-mm no. 1.5 coverslips. Cells were then fixed with 4% PFA in PBS for 15 minutes. For the RNAscope protocol, we followed manufacturer’s instructions for adherent cell lines. Briefly, we first dehydrated the cells using 50%, 70% and then 100% ethanol in PBS. This was followed by a rehydration of the cells using 70% and then 50% of ethanol in PBS. Lastly, cells were fully rehydrated in PBS. Cells were then permeabilized by hydrogen peroxide, followed by protease 3 treatment. Next, we hybridized the RNAscope probes to the cells for 2 hours or O/N at 40C. Probe amplification was then performed, followed by labelling with Opal570 (Akoya Biosciences). Nuclei were stained using Hoechst 33258 (Thermofisher).

For immunofluorescence, cells were fixed, permeabilized by 0.1% Triton X-100 (unless already permeabilized during a prior RNAscope treatment, Sigma Aldrich), blocked with 3% bovine serum albumin (Sigma Aldrich) in PBS. Cells were then immunostained with the indicated antibody, followed by the appropriate secondary. Lastly, nuclei were stained with Hoechst 33258.

Microscopy was performed on an LSM880 with Airyscan (Zeiss, Oberkochen, Germany) or an Olympus (Tokyo, Japan) FV3000RS. On the LSM880, imaging of Huh7-Lunet cells was performed with 20x magnification objective, while NPCs were imaged with a 63x oil objective. On the FV3000RS, the 20x objective was used for imaging NPCs and Huh7-Lunets. All images were taken as a Z-stack.

### Western blot analysis

Cells were lysed in RIPA lysis buffer (50 mM Tris-HCl [pH 8], 150 mM NaCl, 1% NP-40, 0.5% sodium deoxycholate, 0.1% SDS, supplemented with Halt protease inhibitor cocktail [Thermo Fisher Scientific]) to obtain whole-cell lysates or lysed using the NE-PER nuclear and cytoplasmic extraction kit (Thermo Fisher Scientific) to obtain cytoplasmic and nuclear fractions. Proteins were separated by SDS-PAGE and transferred to nitrocellulose membranes (Bio-Rad, Hercules, CA). Proteins were visualized by chemiluminescent detection with ECL and visualized on a ChemiDoc MP Imaging System (Bio-Rad).

### Computational and statistical analyses

For western blot analysis, differences in band intensity were quantified by densitometry using ImageJ [71]. Student’s t-test was used for statistical analysis of western blots. Imaris (Oxford Instruments, Abingdon, UK) was used for analysis of confocal images, using the surface function for polyA mRNA analysis and dot function identifying specific transcripts by RNA-scope. Nuclei were also bounded and identified by the surface function of Imaris. PolyA RNA-scope experiments were statistically analyzed using a linear mixed model to account for individual cell values across multiple biological replicates. Data are represented as means plus standard errors of the means (SEM) as a violin plot produced in Prism 8. Statistical significance was defined as follows: *, P ≤ 0.05; **, P ≤ 0.01; ***, P ≤ 0.001; ****, P ≤ 0.0001. Biological replicates are defined as the same experimental design but performed sequentially, with a different cell passage number and on different days.

### Analysis of RNA sequencing data

PCR duplicates were removed using unique molecular identifiers in the RT primer region. The adaptor and barcode sequences were trimmed and reads were mapped step-wise to viral (ZIKV), repetitive and finally non-repetitive (GRCh38) genomes. Bowtie2 indexes were generated using the ‘bowtie2-build’ command in Bowtie2 for the ZIKV (KU501215.1) RNA genome sequences. The specific parameters used for the FAST-iCLIP pipeline were as follows: -f 18 (trims 17 nt from the 5′ end of the read), -l 16 (includes all reads longer than 16 nt),–bm 29 (minimum MAPQ score from bowtie2 of 29 is required for mapping; unique mapping only),–tr 2,3 (repetitive genome) and–tn 2,3 (non-repetitive genome) RT stop intersection (n,m; where n = replicate number and m = number of unique RT stops required per n replicates). Using the–tr/tn 2,3 parameters, a minimum of six RT stops are required to support any single nucleotide identified as a crosslinking site.

Analysis of the sequencing data was performed using a custom analysis pipeline, with the peak finding software uploaded to Github (https://github.com/ChangLab/FAST-iCLIP/tree/lite). Other analyses were performed by aligning the reads to the human genome using STAR, followed by gene counts using Bedtools [72]. Only reads in the canonical 3’UTR of the human transcripts were counted. The count distribution across the metagene of the CDS and 3’UTRs was visualized using deeptools [73]. Log_2_ fold changes were calculated by comparing RPKM values between Mock and ZIKV infected cells. Genes with fewer than 10 total read counts were excluded from analysis.

Heat maps, XY plots and violin plots were produced using Prism 8 (GraphPad, San Diego, CA).

For total RNA and cytoplasmic/nuclear fractionated sequencing, reads were aligned to the human genome using the STAR aligner [74] (version 2.7.5) followed by HTSeq [75] (version 0.12.3) to obtain counts and then using the DESeq2 [76] (version 1.28.1) pipeline to determine log_2_ fold changes in transcripts. MA plots were produced using DESeq2. Volcano plots were produced using the R-package, Enhanced Volcano (https://github.com/kevinblighe/EnhancedVolcano).

## Supporting information

S1 Fig Electrophoresis of RNA pulled down with UPF1, visualized using the IR handle ligated to the RNAs. The region bounded by the red box indicates the part of the gel excised and then analyzed by irCLIP and AP-MS. AP-MS analysis is indicated in S1 File.
(TIF)

S2 Fig A) Metagenes of the CDS and 3’ UTR created from the RNA seq data of Mock and ZIKV infected NPCs. The graphs show Reads Per Kilobase of transcript, per Million mapped reads (RPKM) values for positions in the Coding Domain Sequence (CDS) and the 3’UTR. Experiment was produced from 2 biological replicates B) MA plot of the 3’UTR from the CLIP data plotting abundance against the fold change. FREM2 has been labeled.
(TIF)

S3 Fig A) Log_2_ fold change from the fractionated RNA-sequencing between the nucleus and cytoplasm for markers of successful fractionation in the mock-infected samples: GAPDH for cytoplasm and ANRIL for nucleus. B) Metascape Analysis of significantly upregulated transcripts found in the bulk RNA sequencing of Fig 2A. The top 400 upregulated genes were used to produce this GO clustering. C) Metascape Analysis of significantly upregulated transcripts found in the cytoplasmic and nuclear fractionated RNA sequencing of Fig 2D. The top 400 and 300 upregulated genes were used to produce this GO clustering.
(TIF)

S4 Fig A) Western blot for UPF1 in siNT and siUPF1 treated Neural Progenitor Cells. Actin is shown as a loading control. N = 1 B) Western blot for UPF1 and Flag in the Tet-inducible UPF1 OE in Lunets. GAPDH is used as a loading control. N = 1.
(TIF)

S5 Fig Individual channels are provided for the microscopy images in Fig 3 corresponding to: A) Fig 3A B) Fig 3B C) Fig 3D D) Fig 3E E) Fig 3F.
(TIF)

S6 Fig Western blot for FREM2 in siNT and siFREM2 treated NPCs after 7 days. Densitometric analyses of FREM2 were performed using ImageJ to quantify relative band intensities. N = 1.
(TIF)

S7 Fig Immunofluorescent staining for markers of differentiation in mock and ZIKV infected NPCs after 48 hours. Quantification performed using the Imaris software suite. N = 15 cells from a single infection. Statistics produced by Student’s t-test. ns–non-significant; ***, P ≤ 0.001. Error bars are SEM.
(TIF)

S8 Fig Individual channels are provided for the microscopy images in Fig 4 corresponding to: A) Fig 4B B) Fig 4D.
(TIF)

S9 Fig Western blots for the siUPF1 experiments measuring FREM2 for Fig 4C. (TIF)

S1 File Slice 1 and Slice 2. Results of Mass Spectrometry Analysis of slices extracted for irCLIP sequencing of ZIKV infected NPCs.
(XLSX)

S2 File Read counts from irCLIP of UPF1 and RPKM of Total RNA Sequencing of ZIKV-infected NPCs and DESeq2 Analysis of the 3’UTR used for the MA plot in S2 Fig. (XLSX)

S3 File DESeq2 Analysis of Total RNA Sequencing of Mock and ZIKV-infected NPCs. (CSV)

S4 File DESeq2 Analysis of Cytoplasmic RNA Sequencing of Mock and ZIKV-infected NPCs. (CSV)

S5 File DESeq2 Analysis of Nuclear RNA Sequencing of Mock and ZIKV-infected NPCs. (CSV)

S6 File Nuclear to cytoplasmic ratios for Fig 3 and data used for graph creation for Figs 3 and 4. (XLSX)
