# Supplementary figures and images for: Nuclear accumulation of host transcripts during Zika Virus Infection
Source: PLoS Pathog. 2023 Jan 5;19(1):e1011070. doi: 10.1371/journal.ppat.1011070 (PMC9847913; doi:10.1371/journal.ppat.1011070)

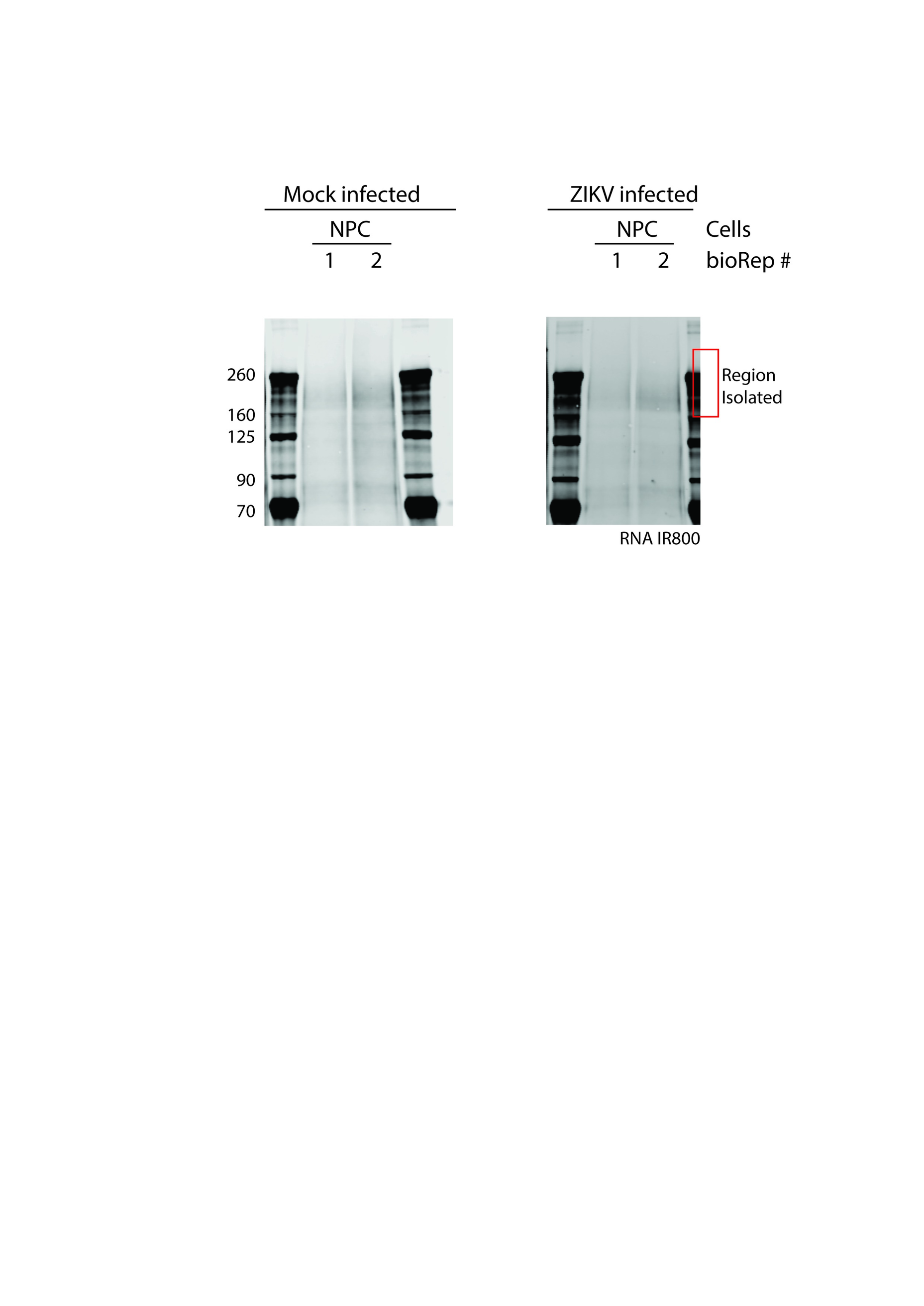

Supplement: S1 Fig — The region bounded by the red box indicates the part of the gel excised and then analyzed by irCLIP and AP-MS. AP-MS analysis is indicated in S1 File. (TIF) [file ppat.1011070.s001.tif]

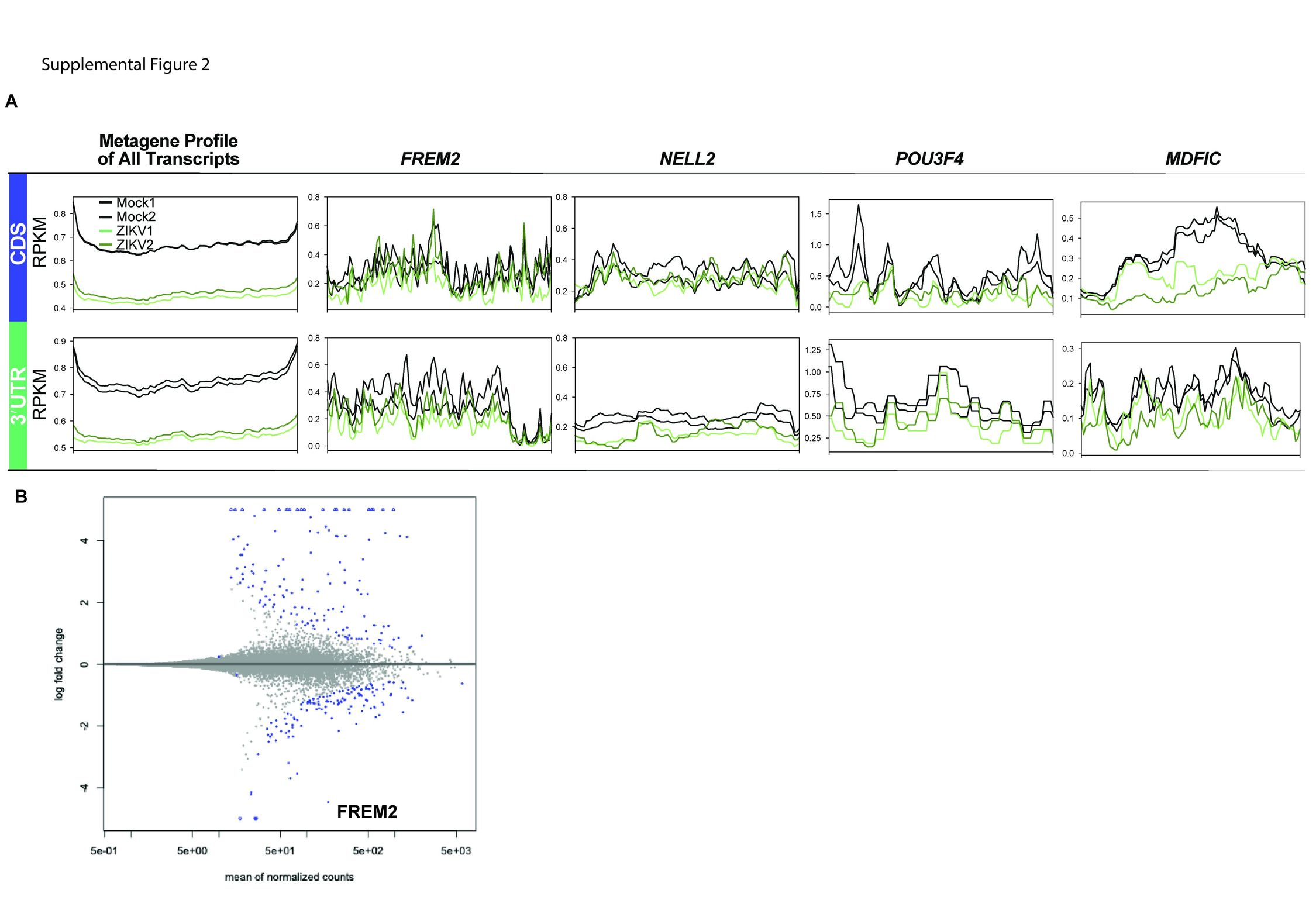

Supplement: S2 Fig — A) Metagenes of the CDS and 3’ UTR created from the RNA seq data of Mock and ZIKV infected NPCs. The graphs show Reads Per Kilobase of transcript, per Million mapped reads (RPKM) values for positions in the Coding Domain Sequence (CDS) and the 3’UTR. Experiment was produced from 2 biological replicates B) MA plot of the 3’UTR from the CLIP data plotting abundance against the fold change. FREM2 has been labeled. (TIF) [file ppat.1011070.s002.tif]

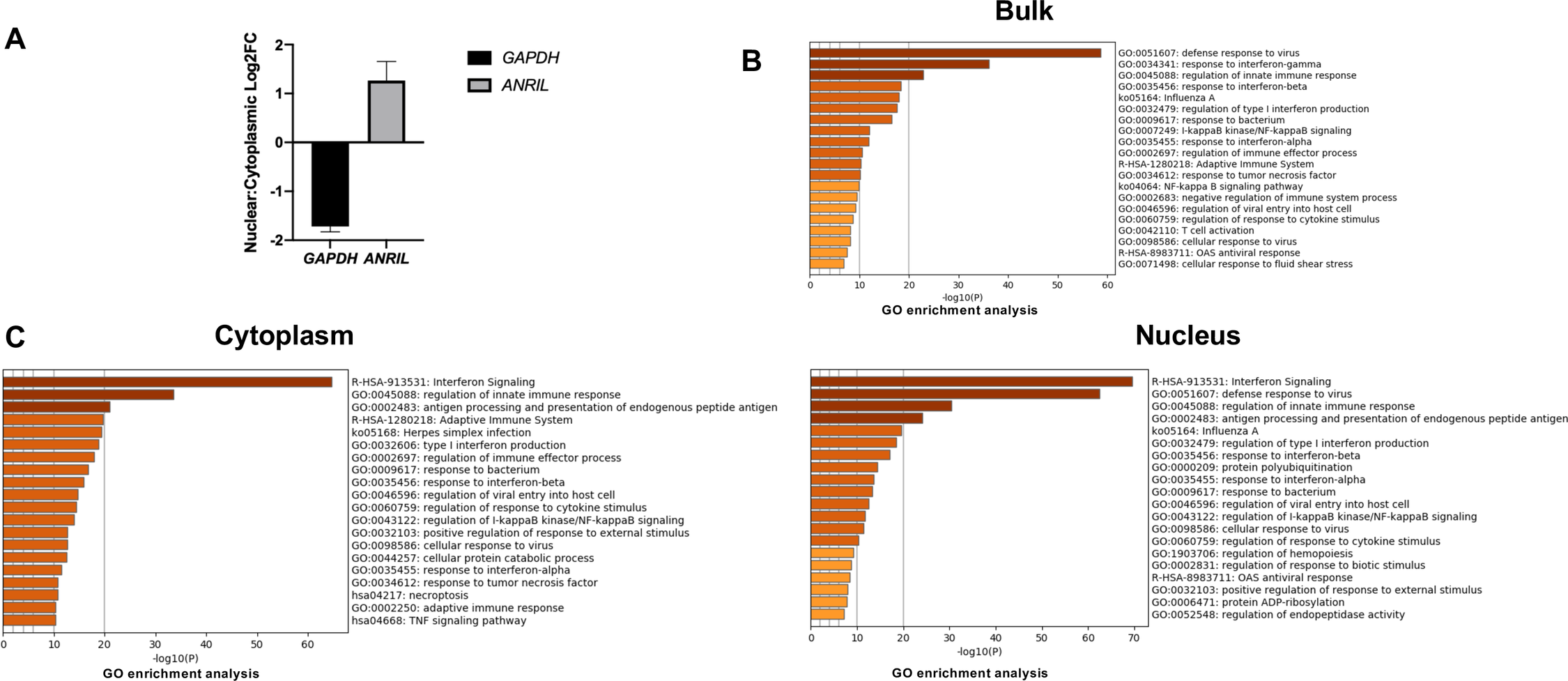

Supplement: S3 Fig — A) Log2 fold change from the fractionated RNA-sequencing between the nucleus and cytoplasm for markers of successful fractionation in the mock-infected samples: GAPDH for cytoplasm and ANRIL for nucleus. B) Metascape Analysis of significantly upregulated transcripts found in the bulk RNA sequencing of Fig 2A. The top 400 upregulated genes were used to produce this GO clustering. C) Metascape Analysis of significantly upregulated transcripts found in the cytoplasmic and nuclear fractionated RNA sequencing of Fig 2D. The top 400 and 300 upregulated genes were used to produce this GO clustering. (TIF) [file ppat.1011070.s003.tif]

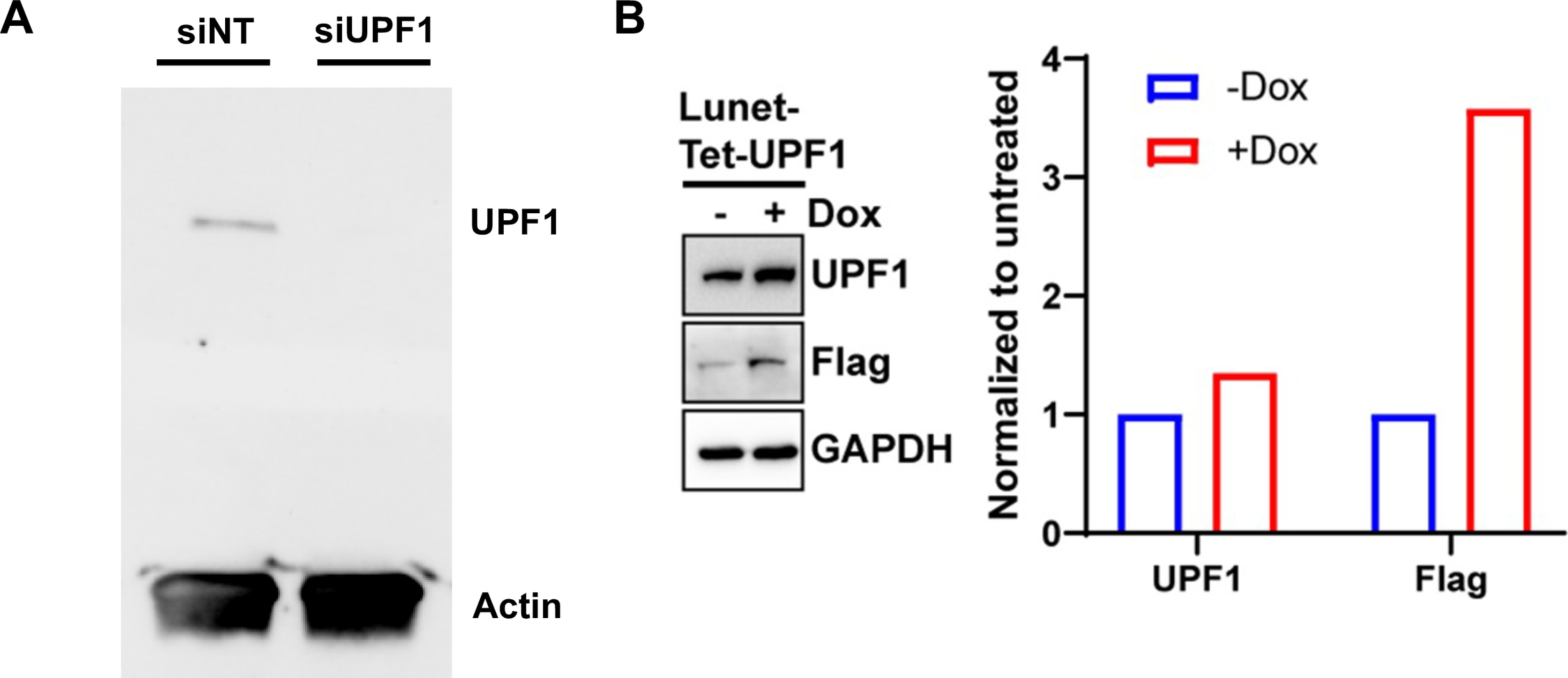

Supplement: S4 Fig — A) Western blot for UPF1 in siNT and siUPF1 treated Neural Progenitor Cells. Actin is shown as a loading control. N = 1 B) Western blot for UPF1 and Flag in the Tet-inducible UPF1 OE in Lunets. GAPDH is used as a loading control. N = 1. (TIF) [file ppat.1011070.s004.tif]

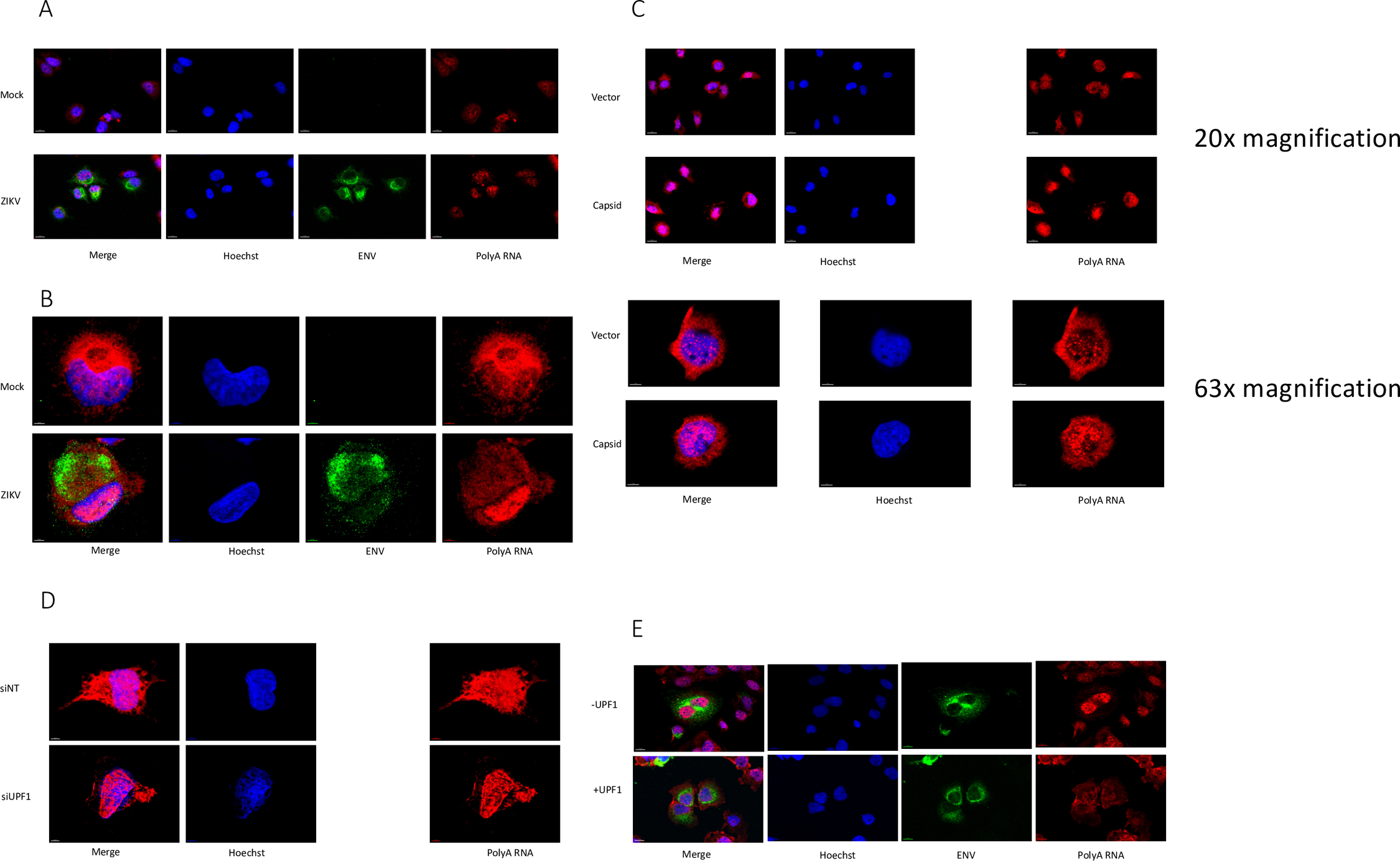

Supplement: S5 Fig — Individual channels are provided for the microscopy images in Fig 3 corresponding to: A) Fig 3A B) Fig 3B C) Fig 3D D) Fig 3E E) Fig 3F. (TIF) [file ppat.1011070.s005.tif]

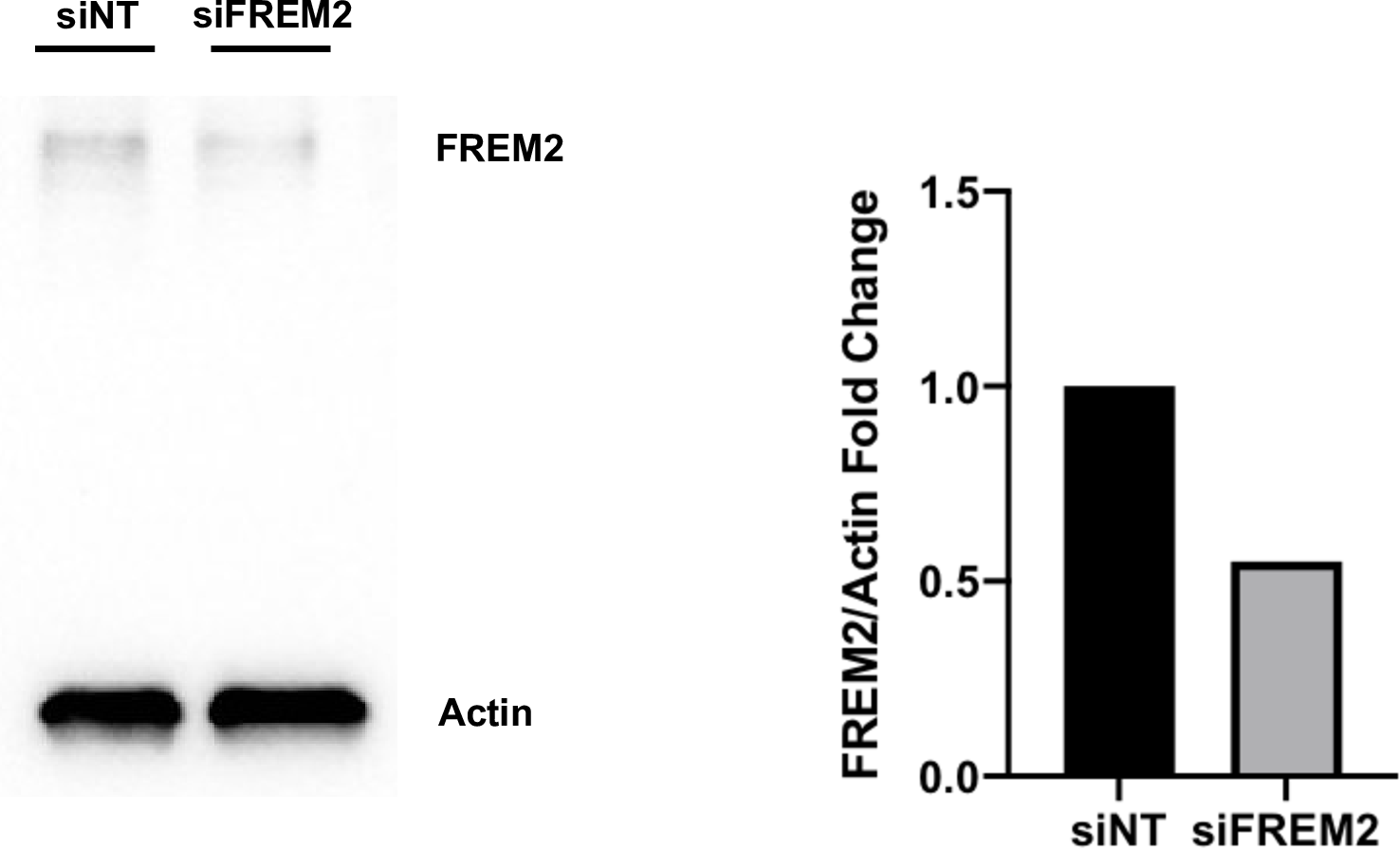

Supplement: S6 Fig — Densitometric analyses of FREM2 were performed using ImageJ to quantify relative band intensities. N = 1. (TIF) [file ppat.1011070.s006.tif]

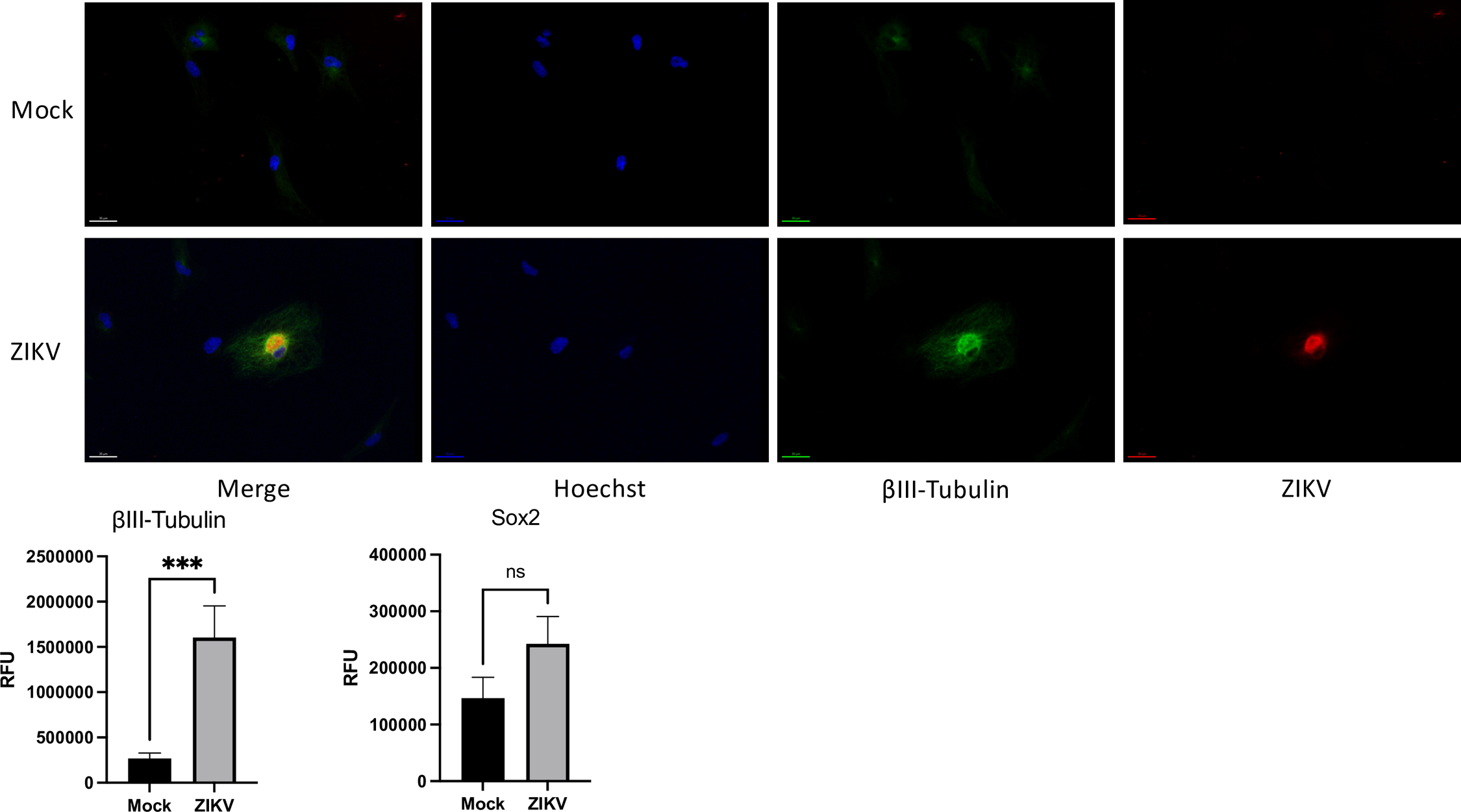

Supplement: S7 Fig — Quantification performed using the Imaris software suite. N = 15 cells from a single infection. Statistics produced by Student’s t-test. ns–non-significant; ***, P ≤ 0.001. Error bars are SEM. (TIF) [file ppat.1011070.s007.tif]

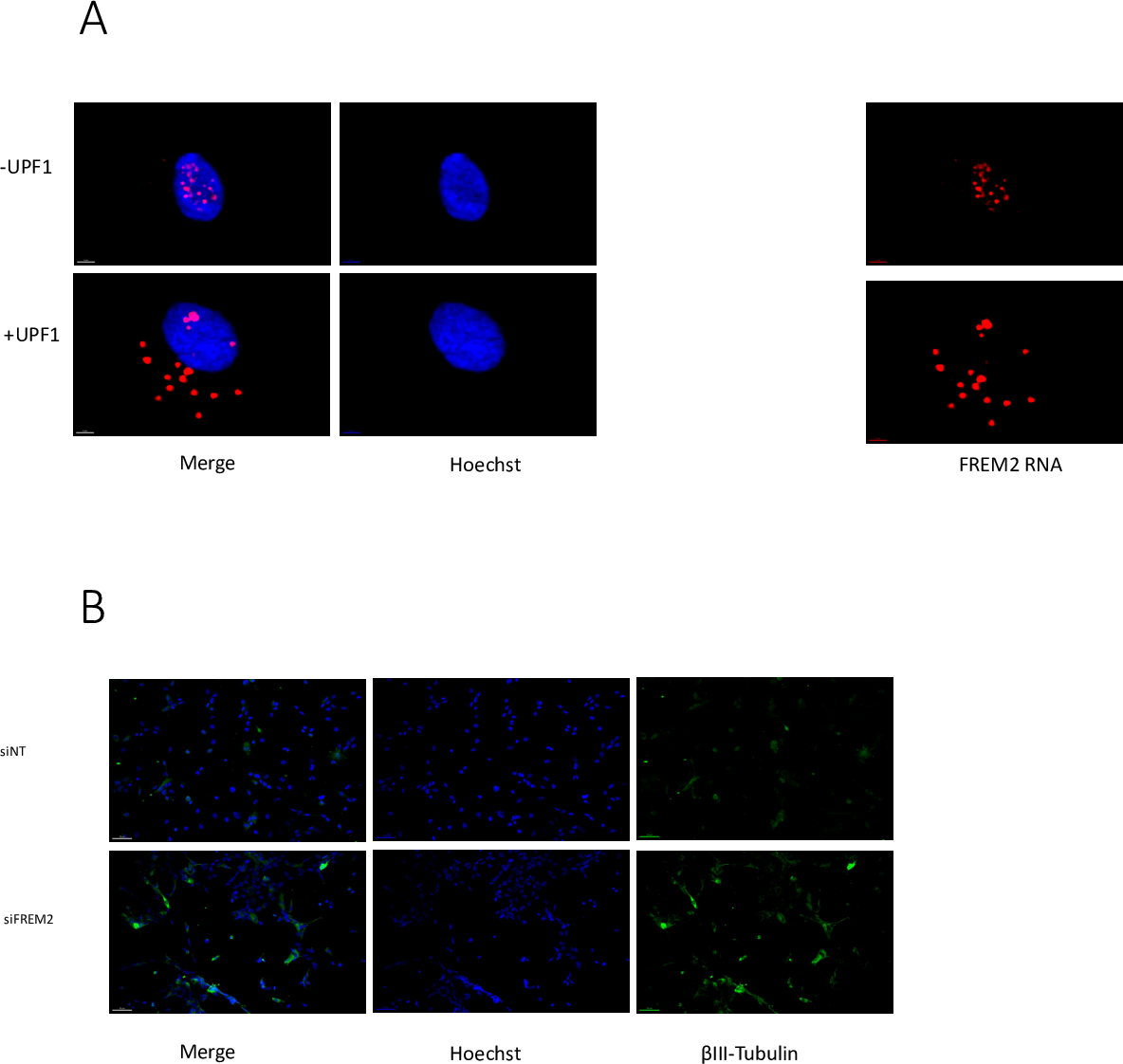

Supplement: S8 Fig — Individual channels are provided for the microscopy images in Fig 4 corresponding to: A) Fig 4B B) Fig 4D. (TIF) [file ppat.1011070.s008.tif]

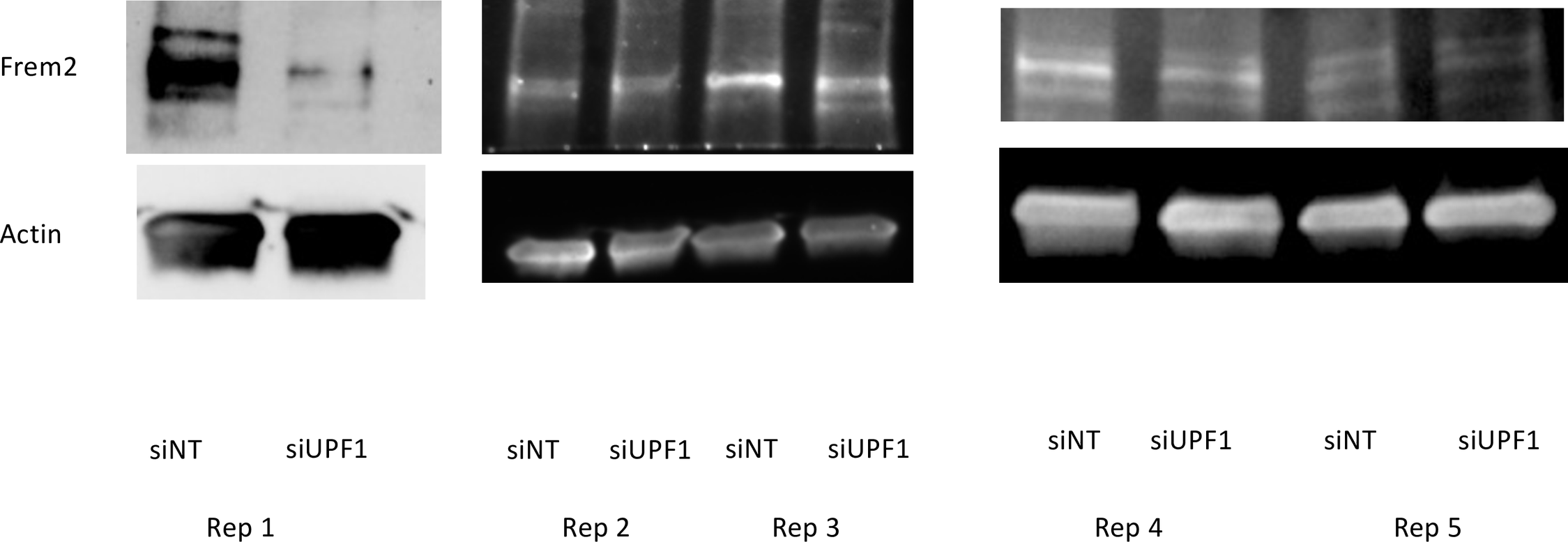

Supplement: S9 Fig — (TIF) [file ppat.1011070.s009.tif]
